# Did the ban on serving raw beef liver in restaurants decrease Enterohemorrhagic *Escherichia coli* infection in Japan?: an interrupted time-series analysis

**DOI:** 10.1186/s12879-019-4576-0

**Published:** 2019-11-08

**Authors:** Kentaro Iwata, Michihiko Goto

**Affiliations:** 1 Division of Infectious Diseases Therapeutics, Kobe University Graduate School of Medicine, Kusunokicho 7-5-2, Chuoku, Kobe, Hyogo 650-0017 Japan; 2 Division of Infectious Diseases, Department of Internal Medicine, University of Iowa Carver College of Medicine, Iowa City, IA 52242 USA; 3 Center for Access and Delivery Research and Evaluation, Iowa City Veterans Affairs Health Care System, Iowa City, Iowa 52246-2208 USA

**Keywords:** Enterohemorrhagic *Escherichia coli*, Hemorrhagic hemolytic syndrome, Interrupted time-series analysis

## Abstract

**Background:**

Enterohemorrhagic *Escherichia coli* (EHEC) is an important pathogen that causes diarrhea, hemorrhagic colitis, and hemolytic uremic syndrome (HUS). After an EHEC outbreak involving uncooked beef, serving raw beef liver dishes at restaurants was completely banned starting on July 1, 2012 in Japan. However, its long-term associations with the incidence rates of EHEC infections have never been assessed by formal interrupted time-series analysis (ITSA).

**Methods:**

A retrospective cohort study to assess the impact of banning raw beef liver provision at restaurants was conducted. The weekly incidence of asymptomatic and symptomatic EHEC infections, the incidence of HUS, and deaths were extracted from the national reportable diseases database from January 2008 to December 2017. ITSA was conducted to evaluate the impact of banning raw beef liver from July 2012. To account for a potential simultaneous external effect, the additional regulation on raw beef red meat handling (implemented in May 2011) and the seasonality were also incorporated into the model.

**Results:**

There were 32,179 asymptomatic and 21,250 symptomatic EHEC infections (including 717 HUS cases and 26 deaths) reported during the study period. During the pre-intervention period (before week 27, 2012), there were 0.45 asymptomatic EHEC infections per million-persons per week. The mean post-intervention asymptomatic EHEC infections were 0.51 per million-persons per week. ITSA revealed no baseline trend or change in the intercept and trend (0.002 infections per million-persons per week, 95% Confidence interval − 0.03-0.04, *p* = 0.93, 1.22, CI -1.96-4.39, *p* = 0.45, and − 0.006, CI -0.003-0.02, *p* = 0.68, respectively). For symptomatic EHEC infections, there were 0.30 cases per million per week during the pre-intervention period, and it became 0.33 cases per million per week after the intervention. Time series modeling again did not show a significant baseline trend or changes in the intercept and trend (0.0005, CI -0.02-0.02, *p* = 0.96, 0.69, CI -1.75-3.12, *p* = 0.58, and − 0.003, CI -0.02-0.01, *p* = 0.76, respectively).

**Conclusion:**

We did not find a statistically significant reduction in the overall incidence rates of both asymptomatic and symptomatic EHEC infections in Japan after implementing measures, including a ban on serving raw beef liver dishes in the restaurant industry.

## Background

Enterohemorrhagic *Escherichia coli* (EHEC) is an important pathogen that causes diarrhea, hemorrhagic colitis, and potentially life-threatening hemolytic uremic syndrome (HUS) [1, 2]. It often causes both sporadic infections and outbreaks worldwide, associated with the consumption of foods contaminated by the organism. EHEC is carried primarily by healthy cattle, young calves and other ruminants [3]. Uncooked or undercooked beef can be contaminated with EHEC during processing and is generally considered to be a major source in most cases. Manure from cattle and other animals can also contaminate produce, including lettuce, fallen fruits, nuts, strawberries, spinach, sprouts, and rocket salad [4–7]. Dairy products can also be a source of infections [7]. Food products such as minced meat cutlet [8], cookie dough or even raw flour can be contaminated and associated with outbreaks [9, 10]. The incidence rates of EHEC infections widely vary internationally, from less than 5 per 100,000-years in South Korea to over 100 per 100,000-years in Iran [11].

The reduction of fecal contamination during slaughter and processing as well as proper cooking are considered as cornerstones in preventing EHEC infections. However, the consumption of raw beef is a part of culinary traditions in many regions globally, and the risk governance to balance public health safety and traditional food culture often causes public controversy.

In April 2011, there was a large outbreak of EHEC strains O111:H8 and O157:H7 in Japan, involving 181 patients with 34 cases of HUS [12]. It was linked to contaminated Yukhoe (Korean-style steak tartare) at franchises of barbecue restaurants [12]. Responding to this outbreak, the Ministry of Health, Labor, and Welfare (MHLW) of the Japanese Government tightened the enforcement of raw beef handling regulations in October 2011 [13]. Subsequently, MHLW also altogether banned serving raw beef liver dishes nationally starting July 1, 2012 with civil and criminal penalties, while strengthening regulations for the testing and processing of raw beef [14, 15]. However, the impact of these measures to reduce the long-term incidence of EHEC infection has never been investigated by an interrupted time-series analysis (ITSA).

The outcome of this Japanese experience can inform public health policymakers in many regions with raw beef dishes as a culinary tradition. In this study, we aimed to evaluate the impact of the nationwide ban on serving raw beef liver dishes on the incidence rates of symptomatic and asymptomatic infections, HUS, and mortality due to EHEC infection by ITSA.

## Methods

### Study population and data source

This is a retrospective cohort study to assess the effectiveness of a nationwide ban on raw beef liver provision at restaurants using interrupted time-series analysis (ITSA). Detection of EHEC from a clinical specimen (either symptomatic or asymptomatic) is a notifiable condition in Japan, and the law requires an immediate report to public health officials after diagnosis. Asymptomatic infection is defined by detection of EHEC from stools of asymptomatic patients, and is not necessarily restricted to outbreak investigation. The law does not specify the required microbiological methods to isolate EHEC, but the screening by selective culture media (Sorbitol-MacConkey agar) is commonly used with genotypic or phenotypic confirmation of verotoxin production. HUS is defined by the presence of the triad of hemolytic anemia, thrombocytopenia, and acute renal failure, and the law requires mandatory reporting if O antigen agglutinating antibody, anti-verotoxin antibody, or verotoxin (either genetically or phenotypically) was detected [16–18]. Local governments collect and submit case-based data via the National Epidemiological Surveillance of Infectious Diseases (NESID) system to MHLW [16]. Data are open to the public and retrievable from the website of the National Institute of Infectious Disease (NIID) in Japan (https://www.niid.go.jp/niid/en/), and we extracted national weekly data from January 2008 to December 2017 for the number of cases of both asymptomatic and symptomatic EHEC infections, of HUS, and death. We calculated cases per million Japanese population based on the annual demographics of Japanese population provided by Statistics Japan (http://www.stat.go.jp/data/jinsui/index.html).

### Intervention

The intervention was enforced by MHLW via amending the Food Sanitation Act of Japan [14]. Effective July 1, 2012, selling beef liver at retail stores or restaurants for raw consumption was banned with civil and criminal penalties. A consumer warning was issued to cook beef liver fully when served for human consumption with specific instructions to heat the core portion of meat to the core temperature at 63 °C for more than 30 min or to use disinfection methods such as heating for 1 min at the core temperature of 75 °C. The law also required retail stores to inform consumers about the need of cooking and provide similar instructions to them. This regulation also applied to beef liver slaughtered and processed before July 2012, after the date of enforcement [14].

### Statistical analysis

Because of the known strong seasonality in incidence rates of EHEC infections [3], we first attempted to extract seasonality and underlying trend components by using additive centered moving average methods, to allow visual inspection of underlying trends throughout the study period [19, 20]. Secondly, to assess and quantify the intervention’s effect, we used segmented linear regression with autoregressive error models, incorporating the baseline trend, an intercept (immediate) change, and a trend change as potential explanatory variables. Seasonality was adjusted by Holt-Winters seasonal smoothing method [21–23], and model appropriateness was assessed by inspecting residual plots. Autocorrelation was assessed by Durbin-Watson statistics and inspecting autocorrelation and partial autocorrelation plots.

We considered a ban on serving raw beef liver as the main intervention and analyzed its effect by single-interruption time-series analysis first, since it was the new regulation which did not exist before July 2012. However, since the tightened enforcement of the preexisting regulation starting October 2011 could have affected incidence rates of EHEC, we also constructed the second model with two interruptions to incorporate both interventions. We also conducted similar analyses using beef consumption per capita in Japan as a denominator. Assuming the enforcement of these regulations took effect immediately after their implementations, we did not include lag effects in these models.

Because of too few cases, we could not apply ITSA to the incidence rates of HUS and mortality. Those data were aggregated to pre-intervention and post-intervention, and we analyzed them as before-and-after fashion by chi-square test. All *p*-values were 2-sided, and alpha = 0.05 was used to set statistical significance.

Stata version 14 (StataCorp, College Station, TX), and R version 3.5.1 (R Foundation for Statistical Computing, Vienna, Austria) were used for all statistical analyses. The ethics committee at Kobe University Graduate School of Medicine exempted this study from the requirement for approval since the study deals with data in the public domain and does not involve individual human subjects.

## Results

**Fig. 1:**
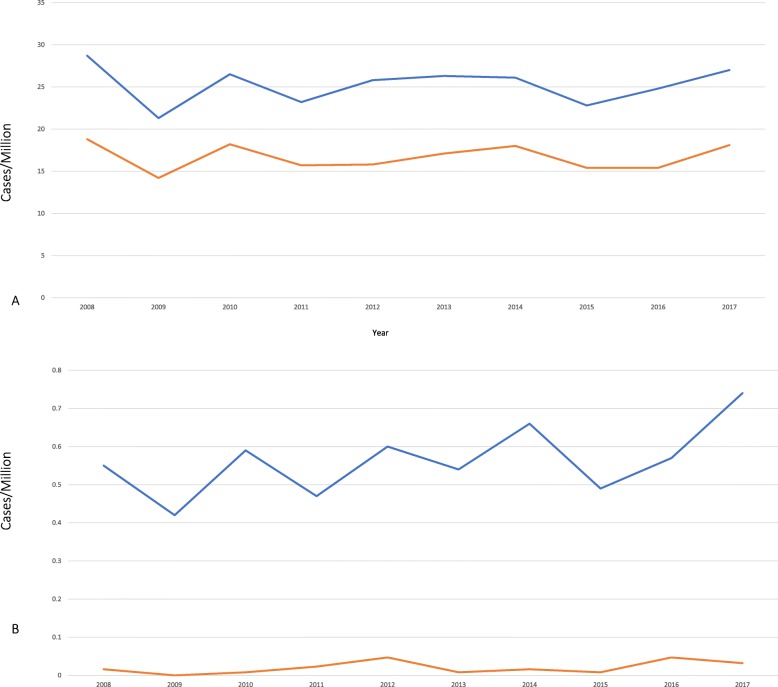
Annual trend of EHEC infections. **a** depicts both asymptomatic and symptomatic EHEC infections reported annually (per million population). **b** describes cases of HUS and deaths per million annually. Abbreviation: EHEC, enterohemorrhagic *Escherichia coli*. HUS, hemolytic uremic syndrome

There were 32,179 (60.2%) asymptomatic and 21,250 (39.8%) symptomatic EHEC infections reported during the study period, including 717 HUS cases and 26 deaths (1.4 and 0.05% respectively among all reported). There was no apparent decrease in each category per million-persons over time (Table 1, Fig. 1).

**Table 1:** EHEC infections in Japan. 2008–2017

Year	Asymptomatic infections	Cases/million	Symptomatic infections	Cases/million	HUS	Cases/million	Death	Death/million
2008	3679	28.7	2403	18.8	71	0.55	2	0.016
2009	2725	21.3	1816	14.2	54	0.42	0	0
2010	3391	26.5	2336	18.2	75	0.59	1	0.008
2011	2965	23.2	2004	15.7	60	0.47	3	0.023
2012	3290	25.8	2016	15.8	76	0.60	6	0.047
2013	3353	26.3	2181	17.1	69	0.54	1	0.008
2014	3320	26.1	2290	18.0	84	0.66	2	0.016
2015	2897	22.8	1957	15.4	62	0.49	1	0.008
2016	3143	24.8	1959	15.4	72	0.57	6	0.047
2017	3416	27.0	2288	18.1	94	0.74	4	0.032
Total	32,179		21,150		717		26	

*Abbreviations*: *EHEC* Enterohemorrhagic *Escherichia coli*, *HUS* Hemolytic uremic syndrome

**Fig. 2:**
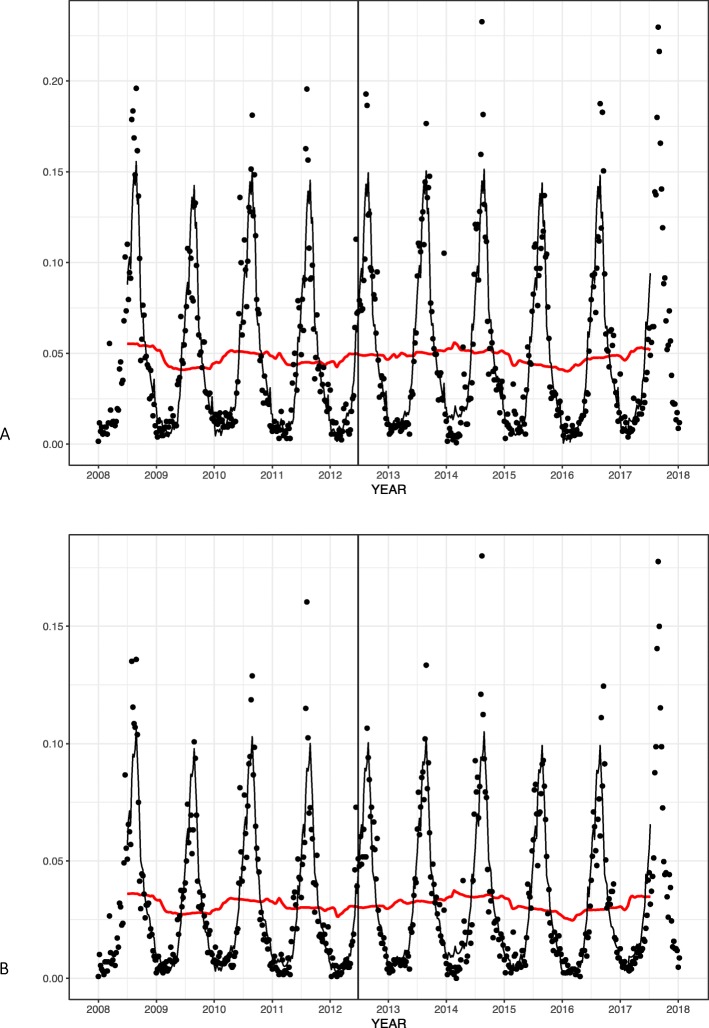
Weekly (**a**) asymptomatic and (**b**) symptomatic EHEC infections in Japan before and after implementing the raw beef liver ban. Black solid lines indicate estimated EHEC cases after adjusting seasonality and red solid lines indicate underlying trends after removing seasonal components. The vertical line indicates the time of intervention

The centralized moving average and deseasonalized trends of the weekly incidence rates of both asymptomatic and symptomatic EHEC infections are shown in Fig. 1.

During the pre-intervention period (before week 27, 2012), the mean incidence rate of asymptomatic EHEC infection was 0.45 per million-persons per week. The mean incidence rate of post-intervention asymptomatic EHEC infections was 0.51 per million-persons per week. ITSA for asymptomatic EHEC showed no significant baseline trend (0.002 infections per million-persons per week. 95% Confidence Interval − 0.03-0.04, *p* = 0.93), intercept change (1.22, CI -1.96-4.39, *p* = 0.45), or post-intervention trend change (− 0.006, CI -0.003-0.02, *p* = 0.68) Fig. 2a.

For symptomatic EHEC infections, the mean incidence rate was 0.30 cases per million per week during the pre-intervention period, and it became 0.33 cases per million per week after the intervention. ITSA again did not show any statistically significant baseline trend, intercept change, and trend change (0.0005, CI -0.02-0.02, *p* = 0.96, 0.69, CI -1.75-3.12, *p* = 0.58, and − 0.003, CI -0.02-0.01, *p* = 0.76, respectively) Fig. 2b. The beef meat consumption of Japanese per capita was largely stable during the study period and the additional analysis with annual beef consumption per capita as the denominator did not show a significant decrease after intervention (data not shown).

The second ITSA analyses with two interruptions (the ban on serving raw liver dishes in July 2012, and the preceding tightened regulation in October 2011) did not indicate a significant intervention effect from either of them, both for asymptomatic and symptomatic EHEC infections (data not shown).

For HUS and mortality due to EHEC, there was no statistically significant change between before and after the main intervention (1.2% vs 1.4%, *p* = 0.07, and 0.03% vs 0.06%, *p* = 0.17 respectively.

## Discussion

The current study did not demonstrate a significant reduction in EHEC infections after implementing a nationwide ban on serving raw beef liver in the restaurant industry in Japan. The incidence rate of EHEC infections in Japan was relatively low among developed countries, even before implementation of the ban. While countries such as Canada, the United States, Australia, the United Kingdom, and the Netherlands have annual incidence rates at > 30 per 100,000 person-years [24], our current study demonstrated that the annual incidence of symptomatic EHEC infections in Japan is less than 20 per 100,000 person-years, and any measure might have a relatively small incremental impact to this already low incidence rate. Also, since a variety of foods such as fruits and vegetables are also associated with EHEC infections, it is unlikely that banning a particular meat or meat product will lead to a significant reduction in incidence, as shown in our study.

Following the interventions analyzed in our study specifically targeting EHEC, the Japanese government continued to implement additional measures responding to sporadic foodborne outbreaks, such as the increased industry regulation for the production of lightly pickled vegetables (Asazuke) in October 2012, or the ban on serving raw pork meats and liver in June 2015 [25, 26]. Each of these interventions caused controversy and attracted attention from the domestic media and the general public, as some of them had popularity among culinary enthusiasts. The risk governance of food safety has always been a delicate balance between traditional culture and public safety, and we believe studies like ours can inform policymakers to create a scientific basis for future regulations and policies. Public health policymakers should conduct further studies to delineate the factors associated with the risk of EHEC infections in Japan and develop strategies which would successfully decrease its risk.

Our study has several limitations. First, only aggregated data for outbreaks and sporadic cases were available, and we could not analyze the effects of interventions to prevent large-scale outbreaks. However, the majority of EHEC cases are sporadic rather than outbreak-associated, and the absolute impact of interventions on outbreak cases should have been even smaller if any [27]. Secondly, because of the lack of detailed weekly data on the Japanese population, we had to use annual data as surrogates, which could potentially compromise the accuracy of our analyses. However, relatively stable population over study period makes it unlikely. Also, the use of autoregressive error models could address potential autoregression introduced by the use of annually-averaged denominator. Thirdly, there might be unaccounted external factors which potentially could have affected the incidence of EHEC, such as climate changes and other non-governmental initiatives in the food industry. Lastly, we were not able to conduct ITSA for HUS and mortality because of their small number of cases, but there was no apparent change in incidence rates before and after the intervention, suggesting that the implementation of the beef liver ban contributed little, if at all, to mitigating both.

## Conclusions

We did not find a significant reduction in the incidence rates of asymptomatic and symptomatic EHEC infections in Japan after implementing a nationwide ban on serving raw beef liver in restaurants. Further study is needed to better guide public health policy to improve food safety including EHEC infections while accounting for culinary traditions and cultures.

## Data Availability

The datasets used and/or analyzed during the current study are available from the corresponding author on reasonable request.

## Abbreviations

EHEC: Enterohemorrhagic *Escherichia coli*
HUS: Hemolytic uremic syndrome
ITSA: Interrupted time-series analysis
